# Development of the chronic obstructive pulmonary disease morning symptom diary (COPD-MSD)

**DOI:** 10.1186/s12955-016-0506-7

**Published:** 2016-07-16

**Authors:** Gary Globe, Brooke Currie, Nancy Kline Leidy, Paul Jones, David Mannino, Fernando Martinez, Paul Klekotka, Sean O’Quinn, Niklas Karlsson, Ingela Wiklund

**Affiliations:** Amgen, One Amgen Center Drive, Thousand Oaks, CA 91320 USA; Evidera, Bethesda, Maryland USA; Institute of Infection and Immunity, St George’s, University of London, London, UK; Department of Preventive Medicine and Environmental Health, University of Kentucky College of Public Health, Lexington, KY USA; Joan and Sandy Weill Department of Medicine, Weill Cornell Medicine, New York, NY USA; Eli Lilly, Indianapolis, IN USA; AstraZeneca, Gaithersburg, MD USA; AstraZeneca, Gothenburg, Sweden

**Keywords:** COPD, Patient-reported outcome, COPD-MSD, Morning, Symptoms

## Abstract

**Background:**

The morning tends to be the most difficult time of day for many patients with chronic obstructive pulmonary disease (COPD) when symptoms can limit one’s ability to perform even simple activities. Morning symptoms have been linked to higher levels of work absenteeism, thereby increasing the already substantial economic burden associated with COPD. A validated patient-reported outcome (PRO) instrument designed to capture morning symptoms will allow for a more comprehensive approach to the evaluation of treatment benefit in COPD clinical trials.

**Methods:**

A qualitative interview study was conducted among a sample of symptomatic adults with COPD. Concept elicitation interviews (*n* = 35) were conducted to identify COPD morning symptoms, followed by cognitive interviews (*n* = 21) to ensure patient comprehension of the items, instructions and response options of the draft COPD Morning Symptom Diary (COPD-MSD). All interview transcript data were coded using ATLAS.ti software for content analysis.

**Results:**

Mean age of the concept elicitation and cognitive interview sample was 65.0 years (±7.5) and 62.3 years (±8.3), respectively. The study sample represented the full range of COPD severity (Global Initiative for Chronic Lung Disease [GOLD] classifications I–IV) and included a mix of racial backgrounds, employment status and educational achievement. During the concept elicitation interviews, the three most frequently reported morning symptoms were shortness of breath (*n* = 35/35; 100 %), phlegm/mucus (*n* = 31/35; 88.6 %), and cough (*n* = 30/35; 85.7 %). A group of clinical and instrument development experts convened to review the concept elicitation data and develop the initial 32-item draft COPD-MSD. Cognitive interviews indicated subjects found the draft COPD-MSD to be comprehensive, clear, and easy to understand. The COPD-MSD underwent minor editorial revisions and streamlining based on cognitive interviews and input from the experts to yield the final 19-item daily diary.

**Conclusions:**

This study supports the content validity of the new COPD-MSD and positions the diary for quantitative psychometric testing.

## Background

Chronic obstructive pulmonary disease (COPD) is a leading cause of death globally despite the fact that it is both preventable and treatable [1]. In addition to high mortality rates, COPD is associated with substantial morbidity as well as economic burden, including direct and indirect healthcare costs [2–4]. COPD is characterized by a number of symptoms, including shortness of breath (SOB), cough, sputum production, wheezing, chest tightness, and fatigue [3]. COPD symptoms are often associated with a decline in functional status and physical activity, and therefore a decline in patient health-related quality of life [5, 6].

Symptoms can vary greatly in severity and frequency and these variations can occur from day-to-day or over the course of a single day [7, 8]. Several studies have shown that, symptomatically, the morning tends to be the most difficult time of day for individuals with COPD and that morning symptoms considerably limit one’s ability to perform simple morning activities [7, 9–11]. Instruments which focus on activities that are integral to daily life are more likely to capture the magnitude of deteriorations and improvements in breathlessness than instruments focusing on nonessential activities [5]. This makes the morning period a particularly good time of the day to ask about activities and symptoms given that certain, basic activities of daily living cannot be avoided in most cases (e.g., getting out of bed, self-care activities). Research has also indicated that patients who experience morning symptoms are at higher risk for exacerbations and are more likely to use their rescue inhaler [11]. Furthermore, morning symptoms, which can be a particular challenge for subjects on the severe end of the disease spectrum, lead to higher levels of work absenteeism for individuals who are gainfully employed [11, 12]. This all points to morning symptoms as an important aggravating factor in the already substantial economic burden associated with COPD.

A few patient-reported outcome (PRO) measures have been developed in recent years to assess COPD patients’ ability to perform morning activities and to evaluate their morning symptoms. For example, the Capacity of Daily Living during the Morning (CDLM) questionnaire and the Global Chest Symptoms Questionnaire (GCSQ) were developed in parallel and as complimentary assessments of morning symptoms and one’s ability to complete activities [13]. Although psychometric testing supported the reliability and responsiveness of both, these questionnaires were developed among a relatively small sample of individuals and saturation of concepts was not expressly demonstrated – an important requirement outlined in the Food and Drug Administration’s (FDA) guidance for the development of PROs [14] – thus, the CDLM and GCSQ may not be appropriate for use in drug development trials to support labeling claims [11, 15]. A relatively new PRO, the Early Morning Symptoms of COPD Instrument (EMSCI), was reported in two abstracts [16, 17]. The EMSCI was designed to measure the frequency and severity of early morning symptoms in subjects with COPD [17]. The EMSCI recently underwent psychometric evaluation in a COPD population enrolled in a Phase 3 clinical trial [16] and although the results suggested good quantitative evaluation properties, the measure is not publically available.

Another available measure of respiratory symptoms in COPD is a derivative of the Exacerbations of Chronic Pulmonary Disease Tool (EXACT), the Evaluating Respiratory Symptoms in COPD (E-RS: COPD) scale. This diary assesses 11 respiratory symptoms of COPD. The development of the E-RS followed good research practices and FDA guidelines for PRO measures [14] and results from clinical trials suggest that the scale is a reliable, valid and responsive measure of COPD symptoms [18, 19]. The EXACT/E-RS is administered daily in the evening to capture respiratory symptoms over a 24-h period without explicit reference to morning symptoms. Although the E-RS is a comprehensive symptom measure, clinical trials may benefit from additional diary questions about morning symptoms for added precision and sensitivity to treatment effects specific to this time of day.

With the morning period being an especially difficult time for some individuals as they experience potentially more frequent and more severe symptoms, having a measure that focuses purely on this sensitive period of the day is an important need that should be met. Thus, given the significance of morning symptoms to patients with COPD, the development of a robust PRO instrument designed to capture morning COPD symptoms in clinical trials as a measure of treatment benefit, with a view to supporting labeling claims, is warranted.

## Methods

**Fig. 1:**
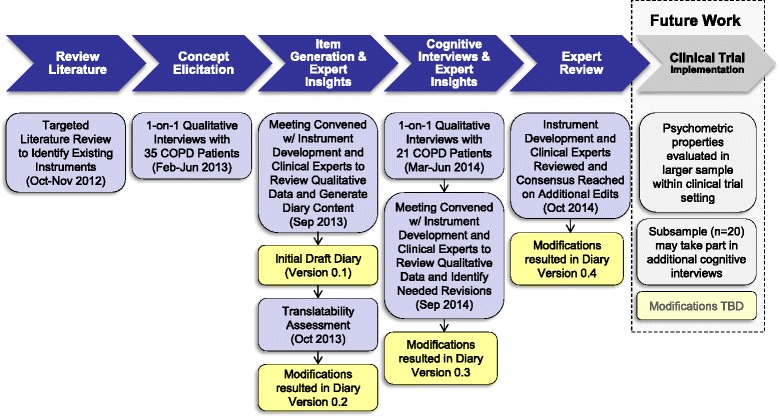
Instrument Development Steps for the COPD Morning Symptom COPD-MSD

An iterative, qualitative process was undertaken to develop the COPD Morning Symptom Diary (COPD-MSD). The process included a review of the literature, which informed the development of an interview guide, input from subjects through qualitative interviews, and discussion with experts (Fig. 1). This paper details the methods, results and conclusions from the two phases of qualitative subject interviews to assure content validity of the final instrument: concept elicitation to capture morning symptom experiences as described by COPD subjects and cognitive interviews to assess patient interpretation of the draft instrument.

Subjects were recruited from 8 pulmonary sites located across the United States (US). Six sites recruited 35 subjects for the concept elicitation phase; 4 sites recruited 21 subjects for the cognitive interview phase. The study protocol (#12198) was reviewed and approved by Ethical & Independent Review Services – a central institutional review board. Written informed consent was obtained from subjects prior to subjects completing any study procedures.

### Inclusion/exclusion criteria for concept elicitation and cognitive interviews

Subjects were included if they met the following criteria: 1) aged ≥40 and ≤75 years at time of screening; 2) diagnosis of COPD with chart-documented post-bronchodilator FEV1/forced vital capacity (FVC) < 0.70; 3) history of cigarette smoking >10 pack years at time of screening; 4) experienced at least one COPD symptom (i.e., cough, shortness of breath, or coughing up mucus/phlegm/sputum) at least 2–3 times a week over the past month that resulted in an impact on the typical morning routine (e.g., getting dressed, moving around the home, preparing breakfast); 5) Modified Medical Research Council (mMRC) Dyspnea Scale score ≥1 at time of screening; 6) history of at least one COPD exacerbation (COPD worsening treated with systemic corticosteroids and/or antibiotics) within the 12 months prior to screening (did NOT apply to subjects classified as Global Initiative for Chronic Obstructive Lung Disease [GOLD] I); 7) ability to read, speak and write English; and 8) were willing to provide written informed consent.

Subjects were excluded if they met the following criteria: 1) primary diagnosis of asthma or any other chronic respiratory disease other than COPD; 2) COPD for any etiology other than tobacco cigarette smoking; 3) congestive heart failure or coronary artery disease with uncontrolled symptoms; 4) treated with chronic systemic corticosteroids as defined by daily use (or equivalent) for at least half of the time in the last year before screening; 5) diagnosis of any other systemic disease considered by the investigator to be clinically significant and unstable/uncontrolled (e.g., Crohn’s disease); 6) participated in a COPD investigational device or drug study within the previous 30 days or planned to begin a new treatment for COPD between the time of screening/enrollment and the study visit; or 7) any other clinically relevant and/or serious concurrent medical condition including, but not limited to visual problems or severe mental illness or cognitive impairment which, in the opinion of the investigator, would interfere with his or her ability to participate in an interview and/or complete the study procedures. Subjects who participated in a concept elicitation interview were excluded from participating in a cognitive interview.

### Measures and methods

Each phase of qualitative interviews involved one study visit in which subjects with COPD participated in a semi-structured interview and completed several self-administered questionnaires including the COPD Assessment Test (CAT), [20] the mMRC Dyspnea Scale, [21, 22] the St. Georges Respiratory Questionnaire for COPD patients (SGRQ-C), [23] and a sociodemographic form. In addition, site clinicians completed a clinical form to capture clinical characteristics for each subject including, but not limited to, date of COPD diagnosis, GOLD stage for airway obstruction, recent pulmonary function testing results (i.e., pre- and post-bronchodilator: forced expiratory volume in one second (FEV1), FEV1 predicted, FVC), number of hospitalizations in past 6 months, key comorbid conditions, and any COPD medications. The clinical form also incorporated the clinician-completed version of the mMRC Dyspnea Scale.

Semi-structured discussion guides were developed – one for the concept elicitation interviews and one for the cognitive interviews – to facilitate general consistency across interviews. Concept elicitation interviews were open-ended, designed to engage the subject in a discussion of their morning symptoms without leading or prompting with specific symptoms. Following this open dialogue, subjects were asked about specific symptoms commonly associated with COPD that were not spontaneously mentioned by the subject. Results formed the basis of a draft instrument tested during cognitive interviews with a new set of subjects. For these interviews, subjects first completed the COPD-MSD in its entirety and then interviewers reviewed the diary questions one-by-one to gather subjects’ interpretation and thought process around their response selection to all items.

### Data analysis

All interviews were audio-recorded and professionally transcribed. The transcripts were entered into ATLAS.ti (version 7.1.7), a software program used for analysis of qualitative research data [24].

Concept elicitation data were reviewed utilizing a content analysis approach to identify themes that described important concepts raised by subjects [25]. Words and phrases provided by subjects to describe symptom concepts were coded and grouped into key themes and relationships. Data from interviews were also analyzed with the goal of comparing and tallying the amount of novel information that was observed in each interview. Interviews were coded in the order in which they were conducted and then grouped sequentially into quartiles [25]. Analysis was conducted to determine whether conceptual saturation–defined as the point at which no new concepts emerge with the addition of more interviews [26–28]—was achieved. A saturation table was created to track emergent information from subjects and analysis was undertaken in a phased approach [29]. Specifically, each new group (quartile) of transcripts was assessed in an ongoing fashion for the appearance of new concept codes [25]. Once no newly appearing codes were documented, saturation was considered achieved.

Following analysis of the concept elicitation transcripts, these data were discussed with clinical experts and instrument development experts, and a draft version of the COPD-MSD was developed, including its instructions, 32 candidate items and response scales. The draft COPD-MSD was then refined based upon feedback from a translatability and readability assessment.

Twenty-one cognitive interviews were conducted with a new sample of COPD subjects using the draft COPD-MSD. Content analyses were also applied to the cognitive interview data, focusing on subject responses to the comprehension of items in the COPD-MSD, salience of the content to their COPD symptom experiences, ease of completion of the COPD-MSD, and subject suggestions regarding item language, instructions, response scales, and formatting. The same experts who participated in the generation of the initial draft COPD-MSD reconvened to review the cognitive interview data and come to a consensus on needed revisions to the COPD-MSD. Two rounds of minor revisions occurred following this meeting, ultimately leading to the COPD-MSD in its current form (see Table 6).

## Results

### Concept elicitation

#### Concept elicitation study sample

The mean age of the concept elicitation sample was 65.0 years (±7.5). Approximately half (*n* = 17, 49 %) were female and a majority (*n* = 28, 80 %) self-identified as white and four subjects (11 %) identified as Hispanic. Subjects represented the full range of COPD severity with 5 subjects (14 %) classified as GOLD I, 11 (31 %) as GOLD II, 11 (31 %) as GOLD III, and 8 (23 %) as GOLD IV. Complete sociodemographic and clinical characteristics are included in Table 1. Subject responses to the PRO measures (CAT, mMRC and SGRQ-C) indicated that the sample generally experienced moderate to high levels of impairment due to COPD (Table 1).

**Table 1:** Subject sociodemographic, clinical, and outcome characteristics

Characteristic^a^	CE (*N* = 35)	CI (*N* = 21)
Age (years)		
Mean, SD, (range)	65.0, 7.5 (51–75)	62.3, 8.3 (44–75)
Gender n (%)		
Female	17 (49)	10 (48)
Racial/Ethnic Background n (%)		
White	28 (87)	13 (62)
Black or African American	4 (11)	4 (19)
Hispanic or Latino	4 (11)	4 (19)
Other^b^	3 (9)	4 (19)
Employment Status n (%)		
Retired	15 (43)	9 (43)
Disabled	9 (26)	8 (38)
Full-time work	6 (17)	1 (5)
Part-time work	3 (9)	2 (10)
Other^c^	2 (7)	1 (5)
Highest Level of Education Completed n (%)		
≤ Secondary/high school	15 (43)	7 (33)
Associate degree	2 (7)	4 (19)
Some college	7 (20)	6 (29)
College degree	9 (26)	2 (10)
Other^d^	2 (7)	2 (10)
Current or Former Smoker n (%)		
Current smoker	15 (43)	3 (14)
Former smoker	20 (57)	18 (86)
Physician-reported GOLD Stage, n (%)		
GOLD I	5 (14)	2 (10)
GOLD II	11 (31)	8 (38)
GOLD III	11 (31)	7 (33)
GOLD IV	8 (23)	4 (19)
Physician-rated mMRC, n (%)		
0	0 (0.0)	0 (0.0)
1	12 (34.3)	7 (33.3)
2	18 (51.4)	5 (23.8)
3	5 (14.3)	7 (33.3)
4	0 (0.0)	2 (9.5)
Patient-reported CAT total score		
Mean, SD (range)^e^	22.0, 6.2 (11–32)	20.1, 8.6 (5–36)
Patient-reported SGRQ-C, mean, SD (range)^f^		
Total score	57.2, 19.9 (4–100)	--
Symptom	71.8, 17.3 (25–100)	--
Activity	66.9, 24.3 (0–100)	--
Impact	46.6, 22.5 (0–99)	--

^a^ All characteristics self-reported by subjects unless otherwise noted

^b^ Other: CE – Chicano (*n* = 1), Hispanic (*n* = 2); CI – Chicano (*n* = 3), Hispanic (*n* = 1)

^c^ Other: CE – Homemaker (*n* = 1); Self-employed (*n* = 1); CI – Unemployed (*n* = 1)

^d^ Other: CE – Postgraduate degree (*n* = 1); 4 years, no degree (*n* = 1); CI – Postgraduate degree (*n* = 2)

^e^ CAT score ranges from 0-40 with scores ≥ 10 indicating the patient is symptomatic and >20 indicating high impairment

^f^ SGRQ total and subscale scores range from 0-100 with higher scores indicating more impairment

#### Concept elicitation findings

##### Morning symptom descriptions and concept saturation

At the outset, subjects were given the opportunity to discuss freely what morning symptoms they experienced as a result of COPD. Prompting and probing from interviewers were minimal at the opening of the interview to allow for as much spontaneous reporting as possible. Following this, interviewers reviewed a checklist of symptoms with subjects to confirm that they experienced no additional morning symptoms related to COPD. In addition to exploring morning symptoms, interviewers asked subjects about sleep disturbance due to COPD symptoms. Some subjects spontaneously mentioned nighttime awakenings prior to the interviewer probing on this experience. Saturation of all concepts was achieved in the first 19 interviews – no new symptom concepts arose in the last 16 interviews that had not arisen in the first 19 interviews (see Table 2).

**Table 2:** Saturation of symptom concepts

	Core Symptom Concepts							Additional Symptom Concepts^a^											
	SOB	Cough	Phlegm	Chest Pain^b^	Wheeze	Trouble Sleeping	Headache	Fatigue^c^	Back Pain	Dizziness^d^	Congestion^e^	Weak Legs^f^	Nasal Drainage	Heart Palpitations	Foot/Ankle Swelling	Dry Mouth	Tight Throat	Numb Face, Lips	Inability to Think, Reason
Group 1 (*n* = 9)																			
102-002	S		P	S	S	P													
102-015	S	S	S	S				S											
102-018	S	S	S	S		P					S		S						
102-028	S	S	S	S		S	S		S	S		S							
102-033	S	S	S	S	S	P		S											
102-035	S	S	S	S	S	P													
104-001	S	S	S		P								S						
104-004	S	S	S	P	P	S	S	S	S			S		S					
104-006	S	S	S		P														
Group 2 (*n* = 9)																			
103-003	S	S	S	S	S														
103-011	S	S	S	S	P		P				S								
104-008	S	S	S	S	S	S	P	S							S				
104-011	S	S	S	P					S			S							
105-001	S	S	S	S	S	P				S									
105-002	S				S		P	S											
105-003	S	S	S					S								S			
105-005	S	S	S	P	P														
105-006	S	S	S	S	P		P												
Group 3 (*n* = 9)																			
103-009	S	S	S		P	P		S		S							S	S	S
103-012	S	S	S		P	S	P												
106-004	S	P	P	S	P	P													
106-006	S						P												
106-007	S	S	P		P	P	P		S										
106-010	S	S	S		S														
106-015	S	S	S	S															
106-016	S																		
106-017	S	S	S	P	S	S													
Group 4 (*n* = 8)																			
102-036	S	P	P																
102-040	S	S	S	P															
102-039	S	S	S	S	P	S	P				S								
102-037	S	S	P	S	S	S													
108-002	S	S	S		P														
108-003	S	P	P					S			S								
108-006	S	S	P	S	P	P	S				S								
108-007	S			P	P		S												
Spontaneous	35	27	24	16	10	7	4	8	4	3	5	3	2	1	1	1	1	1	1
Probed	0	3	7	6	14	9	8	0	0	0	0	0	0	0	0	0	0	0	0
TOTAL (S + P)	35	30	31	22	24	16	12	8	4	3	5	3	2	1	1	1	1	1	1

^a^ These “additional concepts” were *not* included in the symptom checklist used by interviewers, thus these concepts were not probed for by the interviewer rather they spontaneously emerged from subjects

^b^ Includes “chest tightness” and “chest discomfort”

^c^ Includes “exhaustion,” “lack of energy,” “tiredness,” “malaise” and “weakness”

^d^ Includes “lightheadedness”

^e^ Includes “nasal stoppage”

^f^ Includes “legs dragging”

Table 3 presents the most frequently cited morning symptom concepts and is organized by subject quartile with the number of spontaneous versus probed reports noted. The most commonly reported symptom was shortness of breath, which was spontaneously mentioned by all 35 subjects. Other commonly reported (spontaneously and probed) symptoms included: phlegm (*n* = 31, 89 %); cough (*n* = 30, 86 %); wheeze (*n* = 24, 69 %); and chest tightness/discomfort (*n* = 22, 63 %). Other symptoms endorsed by subjects were nighttime awakening/sleep disturbance (*n* = 16, 46 %), headache (*n* = 12, 35 %), and exhaustion/tiredness (*n* = 8, 23 %). Other symptoms were mentioned by subjects, however these were reported with very low frequency (*n* ≤ 5) and were not raised by all four quartiles of subjects; as such, they were not included in Table 3. Forty-two percent (10 out of 24) of the subjects who mentioned wheezing did so without being prompted by the interviewer. A similar proportion of the subjects (7 out of 16) who mentioned nighttime awakening reported it spontaneously. Only one third of subjects who endorsed headaches did so spontaneously. It is important to note that the concept of exhaustion or feeling tired was not included on the interviewer’s symptom checklist, thus all reports of this symptom were spontaneous. Had fatigue or exhaustion been included on the checklist, the total number of reports (probed and spontaneous) likely would have been much higher.

**Table 3:** Most frequently cited morning symptoms by subjects (Concept Elicitation)

	SOB	Phlegm	Cough	Wheeze	Chest Tightness/Discomfort	Nighttime Awakening/Sleep Disturbance	Headache	Exhaustion/Tiredness
Group 1 (*n* = 9)	✓	✓	✓	✓	✓	✓	✓	✓
Group 2 (*n* = 9)	✓	✓	✓	✓	✓	✓	✓	✓
Group 3 (*n* = 9)	✓	✓	✓	✓	✓	✓	✓	✓
Group 4 (*n* = 8)	✓	✓	✓	✓	✓	✓	✓	✓
Spontaneous (S)	35	24	27	10	16	7	4	8
Probed (P)	0	7	3	14	6	9	8	0
Total (S + P)	35	31	30	24	22	16	12	8

##### Subject terminology for morning symptoms

The phrase “shortness of breath” was used spontaneously by 18 subjects (51 %). Other terms included “difficulty/trouble breathing” (*n* = 6), “hard to breathe/can’t hardly breathe/can’t breathe” (*n* = 6), “trouble catching breath/can’t get breath” (*n* = 3), and “out of breath/run out of air” (*n* = 2). Activity level and/or the difficulty or level of effort required to breathe were frequently raised when talking about shortness of breath. More so than any other COPD symptom discussed, it was clear that the severity of a subject’s shortness of breath was closely tied to the type or level of physical activity in which a subject was engaged.

All subjects used the term “cough” when describing the phenomenon of coughing. As subjects spoke about coughing, they generally described the type of cough they experienced (i.e., “heavy,” “hard,” “deep,” “easy,” “dry,” and “productive”). The effort required to cough was frequently mentioned. For all symptoms, but especially so with cough, subjects had a difficult time differentiating between frequency and severity. For example, frequency of cough was directly linked to how subjects viewed the severity of their cough (if one is coughing frequently, their cough was severe). Subjects also discussed the idea of “coughing fits” or “attacks” or relatively intense episodes of cough. Given this feedback, it was later decided that three separate questions would be developed for cough (frequency, severity, and number of coughing attacks) to ensure full coverage of the symptom experience.

Of the 31 subjects who reported experiencing phlegm or mucus, “phlegm” was the most common term spontaneously mentioned with 20 subjects (65 %) using this word. The next most common term spontaneously reported was “mucus” with seven subjects (23 %) using this word. Although “phlegm” was spontaneously mentioned by a majority of subjects, analysis showed that a number of subjects often mentioned both terms and would utilize them interchangeably. Subjects described phlegm in terms of consistency, thickness, and/or color, amount/quantity, and the effort required to cough it up. When analyzing symptom co-occurrence in the transcripts, phlegm was frequently raised by subjects in conjunction with discussion of other symptoms, most notably with cough (225 times), underscoring the proximity of these two symptoms.

Many subjects mentioned the concept of congestion when discussing phlegm which implies that these represent the same or at least closely related concepts to patients. Conversely, several subjects discussed phlegm and congestion as distinct concepts. This informed the development of a separate question in the COPD-MSD for chest congestion.

Twenty-two subjects endorsed experiencing chest tightness or discomfort. Subjects used a variety of words to describe the concept spontaneously with the most common being “chest tightness” (*n* = 12, 55 %). Some subjects would discuss them separately while others used the terms interchangeably. For example, when asked by the interviewer if she/he experienced chest pain or tightness with coughing, one subject commented “tightness but not pain, tightness.” However, other subjects would often dwell on the sense of pain or discomfort associated with breathing or coughing and would link that with tightness. Given the seeming overlap between tightness and discomfort, they were combined for analysis and tallying purposes. It was later decided that a separate question in the COPD-MSD for each concept would be prudent in the early stages of instrument development.

Similar to cough, there were no other words used by subjects to convey the symptom concept of “wheeze.” Of the 24 subjects who endorsed this symptom, all used the term “wheeze.” When discussing this in detail, a number of subjects mentioned hearing their breathing and used words like “whistle,” “squeaky,” and “rattle” to describe the sound.

Of the 16 subjects who discussed having trouble sleeping or waking up in the middle of the night, most expressed difficulty breathing when attempting to sleep and/or waking up due to coughing fits. A number of subjects (*n* = 8) clearly linked trouble sleeping with their COPD symptoms, but a few subjects (*n* = 4) were not certain if their sleep issues were related specifically to their COPD. Several other subjects (*n* = 4) mentioned reasons other than COPD symptoms (e.g., anxiety) as the contributing factor to their sleep disruptions. Eight subjects mentioned feeling a general lack of energy or exhaustion. These were all spontaneous reports as interviewers did not probe for this symptom. Subjects described this concept with a variety of words, including “exhausted,” “wore out,” “malaise,” and “tired/tired out.” Subjects sometimes linked this symptom directly to their interrupted sleep.

##### Morning activities that trigger or worsen symptoms

Subjects were asked to discuss if and how their morning activities impacted their COPD symptoms. Specifically, subjects were asked if any of their morning activities triggered or worsened their symptoms (Table 4). Many varied morning activities (and movements) were mentioned by subjects, however all activities generally fell into 1 of 5 categories: routine indoor physical activity, routine outdoor physical activity, getting oneself ready, general housework, and miscellaneous strenuous activity. Examples of activities that fell into these categories are footnoted in Table 4. Almost all subjects (*n* = 33) spontaneously discussed 1 or more common indoor morning activities or routines that would trigger their COPD symptoms (e.g., going up and down stairs).

**Table 4:** Activities that trigger or worsen COPD symptoms (Concept Elicitation)

	Routine Indoor Physical Activity^a^	Routine Outdoor Physical Activity^b^	Getting Oneself Ready^c^	General Housework^d^	Miscellaneous Strenuous Activity^e^
Group 1 (*n* = 9)	✓	✓	✓	✓	✓
Group 2 (*n* = 9)	✓	✓	✓	✓	✓
Group 3 (*n* = 9)	✓	✓	✓	✓	✓
Group 4 (*n* = 8)	✓	✓		✓	✓
Spontaneous	33	18	11	12	20
Probed	2	7	13	11	0
Total (S + P)	35	25	24	23	20

^a^ Routine indoor activity: going up/down stairs, walking/moving around house, getting out of bed, preparing food/coffee

^b^ Routine outdoor activity: walking dog, picking up newspaper/mail, yard work, sweeping, fishing

^c^ Getting oneself ready: bathing/showering, getting dressed, going to bathroom, putting on socks, tying shoes

^d^ General housework: vacuuming, cleaning, doing laundry, making bed, setting/clearing table

^e^ Miscellaneous strenuous activity: lifting/carrying things, morning exercise (e.g., yoga, elliptical, treadmill)

Subjects were asked to rank the top 3 activities that affected their COPD symptoms in the morning. Routine indoor physical activity typically performed in the morning (e.g., getting out of bed, walking/moving around the home) was ranked as number one more than any other kind of activity (*n* = 14) and also fell into the top three list most often with all subjects (*n* = 32) ranking at least one routine indoor activity somewhere in their top three list. Subjects were then asked which symptoms were impacted by each of their top three activities. Shortness of breath more so than any other symptom was noted by subjects as being affected by their top three activities. This again underscores the close link between physical activity or movement with breathlessness (data not shown).

### Item generation

Based upon close review of these qualitative data, a draft version of the COPD-MSD, including its instructions, 32 candidate questions, and response scales was developed by a group of clinical experts and instrument development experts who convened in September 2013. Key discussion and decision points that informed the COPD-MSD’s content are included in Table 5. The draft was reviewed by a translation and cultural specialist and edits were made for clarity and translatability. The revised draft COPD-MSD was assessed in a new sample of 21 COPD subjects using cognitive interview methodologies.

**Table 5:** Key notes from item generation expert panel meeting (September 2013)

Notes/Group Decisions
• It is difficult for subjects to link nighttime awakenings to symptoms, thus do not ask subjects to report number of awakenings related to symptoms; one advisor suggested adding a proxy item to capture nighttime awakenings (“How rested did you feel this morning?”) – this item would be in addition to a question regarding how tired or fatigued the subject felt
• All symptom concepts, including those with relatively low subject endorsement (e.g., headaches, exhaustion/tiredness), should be included; advisors agreed to err on side of inclusivity in the early stages of instrument development. It was noted items that perform poorly would drop out in the future based on psychometric assessments
• Wheeze was debated at length among advisors with some noting it was a difficult symptom to measure, however advisors agreed to include wheeze for now with potential for deleting later
• Although unclear if “chest congestion” was viewed by subjects as a distinct symptom from phlegm or other chest symptoms (i.e., tightness, discomfort), include as separate item for now
• For those miscellaneous symptoms that were mentioned infrequently (*n* < 5) and were not clearly related to COPD (e.g., back pain), advisors agreed to exclude from COPD-MSD
• Shortness of breath (but not other symptoms) should be measured in the context of performing activities, however need to select activities that are relatively universal; advisors recommended reviewing literature and exploring this with subjects in cognitive interviews
• Advisors concluded a Likert scale would be best suited for the COPD-MSD items
• Key questions or themes to explore during cognitive interview phase:
o Explore subject language used around the “tiredness/exhaustion” concept as well as how subjects describe and define “chest congestion” (i.e., is chest congestion different from chest tightness and chest discomfort?)
o Explore what morning activities are universal or common to all subjects
• For recall period, ask about morning timeframe in general, do not limit or narrow to a specific hour within the morning period

### Cognitive interview

For brevity, the cognitive interview results presented below relate only to the content that ultimately was retained in the final 19-item version of the COPD-MSD (Table 6).

**Table 6:** COPD-MSD items and response scales

	COPD Item	Response Range^a^
Items asking about “this morning”		
AM 1	SOB upon getting out of bed	No SOB → Unable to because of SOB
AM 2	SOB while washing self	No SOB → Unable to because of SOB
AM 3	SOB while using arms to do things	No SOB → Unable to because of SOB
AM 4	SOB while getting dressed	No SOB → Unable to because of SOB
AM 5	SOB while bending over	No SOB → Unable to because of SOB
AM 6	SOB upon moving around the home	No SOB → Unable to because of SOB
AM 7	Coughing attacks	0 → More than 5
AM 8	Frequency of cough	Not at all → Almost constantly
AM 9	Severity of cough	Did not cough → Very severe
AM 10	Amount of phlegm	None → Very large amount
AM 11	Difficulty bringing up phlegm	Did not have phlegm → Very difficult
AM 12	Severity of chest tightness	No chest tightness → Very severe
AM 13	Severity of chest discomfort	No chest discomfort → Very severe
AM 14	Severity of chest congestion	No chest congestion → Very severe
AM 15	Severity/amount of wheezing	Not at all → Very much
AM 16	Feeling tired	Not at all → Extremely
AM 17	Feeling rested	Not at all → Extremely
Items asking about “last night, after going to bed”		
PM 1	Severity/amount of wheezing	Not at all → Very much
PM 2	Sleep disturbance	Not at all → Very much

Abbreviations: *COPD-MSD* Chronic Obstructive Pulmonary Disease – Morning Symptom Diary, *SOB* shortness of breath

^a^ The six SOB items utilize a 6-point response scale while all other items use a 5-point range

#### Cognitive interview study sample

The mean age of the cognitive interview sample was 62.3 years (±8.3). Half were female (*n* = 10, 48 %) and a majority (*n* = 13, 62 %) identified as white. Subjects represented the full range of COPD severity with two subjects (10 %) classified as GOLD I, eight subjects (38 %) as GOLD II, seven subjects (33 %) as GOLD III, and four (19 %) classified as GOLD IV. Complete sociodemographic and clinical characteristics are in Table 1. Subject responses to the PRO measures (CAT, mMRC) indicate that these subjects had moderate to high impairment due to their COPD (Table 1). To alleviate subject burden and shorten the study visit, cognitive interview subjects were not administered the SGRQ-C.

#### Cognitive interview findings

Overall, subjects reported that the COPD-MSD covered their full range of morning COPD symptoms and was easy to complete. On average, subjects completed the 32-item COPD-MSD within 8 min. A majority (*n* = 18, 86 %) said the COPD-MSD was relevant to and covered their morning symptoms. Seven subjects (33 %) recommended adding one or two items, including: a question on strength of cough (e.g., how violent are the coughing attacks), and dry mouth, and a specific question on level of exertion or physical activity (e.g., walking up hill), and question about the impact of COPD symptoms (e.g., sexual activity). Importantly, no new symptom concepts emerged from the cognitive interviews, further supporting that conceptual saturation had been achieved in the concept elicitation interview phase.

##### Instructions and recall period

A majority of subjects (*n* = 17, 81 %) understood and followed the COPD-MSD’s morning instructions. Two subjects (10 %) recommended changing the recall period to the past week, stating that a week would be a better evaluation period of their good and bad days with symptoms.

##### Shortness of breath items

All subjects (*n* = 21, 100 %) reported that at least one of the activities of the seven shortness of breath items triggered breathlessness. Bending over (*n* = 19, 91 %), moving around the home (*n* = 17, 81 %), getting dressed (*n* = 16, 76 %), and making the bed (*n* = 15, 71 %) were the most commonly reported activities that triggered shortness of breath. The activities in the shortness of breath items generally were reported as being a part of subjects’ typical morning routine. However, one subject (5 %) bathed at night rather than the morning and one subject was confined to a wheelchair which influenced how he/she answered these questions and thought about physical activities in general. Approximately two thirds of subjects (*n* = 14, 67 %) stated that making the bed was a typical part of their routine, while seven subjects (33 %) said that it was not and/or that their partner would usually make the bed. Of the latter, a majority were men (*n* = 5) indicating a potential gender bias regarding responsibilities around making the bed. Two subjects (10 %) reported that they were unable to make their bed due to shortness of breath. Note the item on making the bed (*How severe was your shortness of breath while****making the bed****this morning?*) was removed following the cognitive interviews and discussion with clinical and instrument experts*.*

When talking about their morning activities, a majority of subjects (*n* = 17, 80 %) described how two or more of the seven shortness of breath activities overlapped with one another. For example, “bending over” and “getting dressed” were the activities most commonly mentioned in conjunction with one another. Subjects typically stated that they would bend over to put their socks and shoes on. “Using your arms to do things” was another activity that subjects commonly reported in connection with other morning activities, including “making the bed,” “getting dressed,” and “washing.” Several subjects (*n* = 4, 19 %) said they avoided bending over to prevent triggering their shortness of breath.

Subjects recommended adding examples to help clarify a few of the morning activity items. A majority of subjects (*n* = 13, 62 %) suggested adding an example for the “using your arms to do things” item, including: reaching arms above head, cooking, and lifting objects. Approximately half of the sample (*n* = 10, 48 %) suggested adding an example for the item about bending over and several subjects (*n* = 3, 14 %) suggested including an example for “moving around the home.”

##### Cough items

Nearly all subjects (*n* = 19, 90 %) understood the cough items. One subject (5 %) had difficulty differentiating between duration and frequency of their cough and suggested defining the duration of a coughing attack. Most subjects described the phrase “coughing attack” as a constant and persistent cough. A few subjects (*n* = 3, 14 %) suggested changing the term “coughing attack” to “constant coughing” or just “coughing.”

A majority of subjects (*n* = 13, 62 %) viewed the item on coughing attacks and the item on how often one coughed as asking about different concepts. Similarly, a majority of subjects (*n* = 16, 76 %) viewed the items “how often did you cough” and “how severe was your cough” as questions about different concepts.

##### Phlegm/mucus items

All subjects (*n* = 21, 100 %) demonstrated an understanding of the phlegm items by discussing what each item meant based on their personal experiences. Almost all subjects (*n* = 18, 86 %) viewed these items as asking about different concepts.

##### Chest symptom items

All subjects (*n* = 21, 100 %) understood the chest symptom items. Notably, over half of the sample (*n* = 14, 67 %) said at least two of the four chest items were asking about the same symptom with nine subjects (43 %) stating that chest tightness and chest discomfort represented the same issue.

##### Tired and rested items

All subjects (*n* = 21, 100 %) demonstrated an understanding of the tired and rested items, though over half the sample (*n* = 12, 57 %) reported that the two items were asking about the same concept.

##### Nighttime instructions and recall period

Almost all subjects (*n* = 18, 86 %) understood the nighttime instructions, stating the section was asking them to think about symptoms after going to bed. Several subjects (*n* = 4, 19 %) mentioned that the instructions were unnecessary given that the items themselves clearly stated the recall period.

##### Nighttime items

All subjects (*n* = 21, 100 %) understood the intended meaning of the nighttime wheeze item and nearly all subjects (*n* = 19, 90 %) understood the sleep disturbance item. Two subjects (10 %) thought the phrase “lung symptoms” encompassed COPD symptoms as well as other respiratory conditions such as lung cancer. A few subjects (*n* = 3, 14 %) mentioned using oxygen at night and two of these subjects specifically stated that this alleviated their symptoms (i.e., impacted the way they responded to the item).

##### Response options

Overall, subjects understood the response options for all COPD-MSD items and could identify that they represented scales of increasing intensity or severity. A number of subjects seemed to struggle with differentiating between at least two response choices, most frequently at the more severe end of the scale. For example, about a third of subjects (*n* = 8, 38 %) stated that the options “severe” and “very severe” were the same when discussing the cough severity item. Another example of this was demonstrated with the tired and rested items where eight subjects (38 %) said that “very” and “extremely” had the same meaning for these items. For the wheeze item, subjects struggled to differentiate between options in the middle of the scale with nine subjects (43 %) stating “a little” and “somewhat” were the same. The number of subjects who struggled with differentiating between response options varied by the diary item and response scale.

##### Changes made to COPD-MSD following data review by clinical and instrument experts

Issues noted by subjects were flagged for discussion with clinical experts and instrument development advisors. Several changes were made to the COPD-MSD following close review and discussion of the cognitive interview data. These changes are summarized in the paragraphs that follow.

1. It was initially concluded by the item generation team of experts that the early version of the COPD-MSD should err on the side of inclusivity rather than exclusivity, thus the version of the COPD-MSD that was tested in the cognitive interviews included questions related to both morning and nighttime symptoms. Following the cognitive interviews, it was clearer that the nighttime symptoms, while important to subjects, did not appear to be interrelated to their morning symptoms. While this left the opportunity to develop two separate measures, it was not consistent with the key aim of developing the COPD-MSD (i.e., assessment of morning symptoms) and so most nighttime items were removed with the exception of an item on nighttime wheeze and one on general sleep disturbance. The nighttime wheeze and sleep disturbance items were retained for further study, based on recommendations from clinical advisors. Specifically, nighttime wheezing was discussed as being a particularly important or challenging symptom for subjects based on findings from a large observational study conducted by Kessler et al. [7], thus advisors suggested retaining this item and tracking its performance in future studies.
2. Given the lack of consistency and potential gender bias around making the bed in the morning, this item was removed but “making the bed” was added as a parenthetical example in the “using your arms to do things” item.
3. It was determined that headache was not endorsed sufficiently enough by subjects nor was it linked consistently to COPD; therefore the two items related to headache were removed.
4. The version of the COPD-MSD that was tested with subjects in the cognitive interviews included two global questions and several items on use of short-acting inhalers. It was decided that these items were not necessary or appropriate to maintain as part of the final version of the COPD-MSD. Global items will be administered separately from the COPD-MSD when it comes time to evaluate the psychometric properties of the COPD-MSD. Similarly, questions on short-acting inhaler use will be developed and administered separately from but in conjunction with the COPD-MSD in future clinical trial settings.

Based on results of the cognitive interviews, the COPD-MSD was significantly streamlined, reducing the number of items from 32 to 19.

## Discussion

This paper describes the methods and results of qualitative research conducted to assure content validity of the COPD-MSD, a new daily diary for evaluating morning symptoms of COPD. A review of the literature confirmed the need for this instrument and informed the development of an interview guide for concept elicitation interviews during which patients with COPD were asked to describe the type and nature of their morning symptoms. Key symptoms reported by study subjects included shortness of breath with activity, cough, phlegm, chest tightness, chest congestion, chest discomfort, wheeze, fatigue, and impaired sleep quality. These symptoms align with those identified in the literature [2, 4, 8, 30, 31] and as outlined by the most recent GOLD report [3]. A 32-item draft version of the COPD-MSD was tested in a new set of COPD subjects using cognitive interview methodology. Based on these patient insights and input from clinical experts and instrument development advisors, the diary was refined to yield the final 19-item COPD-MSD.

The prevalence of morning symptoms and the impact of those symptoms on subjects’ ability to perform routine morning activities was confirmed in the study interviews and are in agreement with findings and conclusions drawn by other researchers in this area [7, 9, 10] and thus supported the decision to develop an instrument that focused on the morning symptom experience in this subject population. The recent development and psychometric validation of the EMSCI which assesses early morning symptoms and impact also demonstrates a growing interest and emphasis on this critical time of day for COPD subjects. The items resulting from this qualitative work closely mirror symptoms captured by the E-RS scale suggesting that these truly represent the symptoms most important to COPD subjects. The new COPD-MSD instrument possibly could be administered as a complimentary measure to the EXACT/E-RS [18, 19] in a clinical trial setting with the COPD-MSD being used to measure the morning symptom experience and the E-RS each evening to capture subject symptoms over the previous 24 h period.

As evidenced by input from subjects, there appeared to be some conceptual overlap between the “tired” and “rested” questions. The “How tired did you feel this morning?” item was driven by insights collected during the concept elicitation interviews and was based on subjects who reported feeling tired, fatigued or exhausted in the mornings due to their COPD symptoms. The “How rested did you feel this morning?” item was added following a discussion with advisors about how best to capture nighttime awakenings. One advisor commented that it is difficult for subjects to link awakenings to specific symptoms and therefore suggested asking about how well rested subjects felt as a proxy for nighttime awakenings. It is likely that one of these items will be removed based upon quantitative results from a larger, psychometric validation study (i.e., items will likely demonstrate high inter-item correlations and confirm redundancy in concept coverage).

An interesting finding was related to patient descriptions of chest symptoms and their use of terminology to describe these experiences. Subjects used terms such as “chest tightness,” “chest discomfort,” “chest congestion,” “phlegm,” and “wheeze” with a personal understanding of each. Some subjects used these terms interchangeably, while others had a unique definition for each. Wheeze was described as a sound or noise, with varying interpretation. This symptom may be more relevant and interpretable to patients with asthma or those with COPD and reversible airflow obstruction. Quantitative data are needed to examine these items further, including their individual performance properties, inter-item correlations, factor structure, and relationship to socioeconomic and clinical characteristics. Based on these analyses, it may be appropriate to drop one or more of the items.

Three subjects (14 %) described how taking their rescue inhaler or nebulizer in the morning would reduce the severity of their shortness of breath as their morning routine progressed. Even though the impact of taking medication in the morning was only mentioned by a few subjects, these comments could have important implications for future clinical trial implementation and administration of the COPD-MSD. Specifically, the timing around use of the morning inhaler as well as the timing of COPD-MSD administration needs to be carefully conveyed in order to avoid introducing bias that could affect subjects’ responses to the COPD-MSD. Several subjects commented that their nighttime oxygen treatment would or did affect how they responded to the COPD-MSD, suggesting this will also need to be addressed in clinical trial implementation and logistics.

Subjects who took part in these concept elicitation and cognitive interviews represented the complete range of COPD severity (GOLD I–IV), varying levels of educational achievement, race and ethnicity, and both genders were equally represented. However, it should be noted that these interviews were conducted among subjects who had a history of smoking and who had been relatively symptomatic at the time of screening for the study. The relevance and ability of the COPD-MSD to fully capture the experience of never smokers or individuals who are less symptomatic has yet to be evaluated.

The main limitation of this study was that only a single round of cognitive interviews was conducted. The instrument development process would have been strengthened by an additional round of cognitive interviews among a small sample of COPD subjects to confirm that the streamlined, 19-item COPD-MSD was accepted and understood by these subjects. Additional interviews also may have helped to further clarify patient perceptions of chest tightness and discomfort, chest congestion and phlegm.

The next phase of development and validation for the COPD-MSD will involve the application of quantitative methods to reduce the length of the instrument, including item-level descriptive statistics (floor and ceiling effects, level of missingness), item-to-item and item-to-total correlations, and the use of Rasch and factor analyses. With the instrument content and structure finalized, COPD-MSD scores will be tested for reliability, validity, and responsiveness to change. These analyses could be performed in a separate observational validation study or through secondary analyses of data from a clinical trial.

## Conclusions

Research indicates that mornings tend to be the most symptomatic period of the day for patients with COPD. A measurement tool that can quantify this experience could be useful for understanding the benefit of new medicines. Qualitative research involving patients with COPD and input from clinical and measurement experts yielded the 19-item COPD-MSD, ready for quantitative testing.

## Abbreviations

CAT, COPD assessment test; CDLM, capacity of daily living during the morning; COPD, chronic obstructive pulmonary disease; COPD-MSD, COPD morning symptom diary; EMSCI, early morning symptoms of COPD instrument; E-RS, EXACT-respiratory symptoms; EXACT, exacerbations of chronic pulmonary disease tool; FDA, food and drug administration’s; FEV1, forced expiratory volume in one second; FVC, forced vital capacity; GCSQ, Global Chest Symptoms Questionnaire; GOLD, Global Initiative for Chronic Obstructive Lung Disease; mMRC, Modified Medical Research Council Dyspnea Scale; PRO, patient-reported outcome; SGRQ-C, St. Georges Respiratory Questionnaire for COPD patients
